# Recent Progress on the Applications of Nanomaterials and Nano-Characterization Techniques in Endodontics: A Review

**DOI:** 10.3390/ma15155109

**Published:** 2022-07-22

**Authors:** Olcay Özdemir, Turkan Kopac

**Affiliations:** 1 Department of Endodontics, Faculty of Dentistry, Karabük University, 78050 Karabük, Turkey; 2 Department of Chemistry, Zonguldak Bülent Ecevit University, 67100 Zonguldak, Turkey

**Keywords:** nanotechnology, nanomaterials, nano-characterization techniques, nano-dentistry, endodontics, nano-endodontology

## Abstract

The impact of nano-based technologies in endodontics for the identification and treatment of various dental infections is showing fast progress. Studies show that nanoparticles could serve as useful agents with many beneficial results and continue to be promising in the field of endodontics. To ensure progress and improvements on novel nanomaterials in relation to their physicochemical and biological properties, nano-identification methods for the detection and evaluation of diseases need to be further highlighted. This study aims to review the current technological progress and recent research outcomes as well as possible prospective applications of nano-based technologies in endodontics. A comprehensive literature survey has been carried out on the utilizations of nanomaterials and nano-characterization techniques in endodontics. The current status and recent applications in endodontics are discussed with illustrative examples. The results have shown that the progress and improved accuracy of nano-identification techniques enabled a better characterization, evaluation and selection of appropriate treatment plans for endodontics-related diseases. The results have been inspiring for further clinical investigations. Nano-endodontics is still a developing field with a strong potential for revolutions of novel materials and techniques in the diagnosis and treatment of dental diseases. Further improvements in nanoparticles properties will pave the way for the development of many beneficial endodontic therapeutic agents. The future looks encouraging for sustainable products and testing methods for clinical endodontic applications.

## 1. Introduction

The term nanotechnology involves the science, technology and engineering studies at nanoscale dimensions, corresponding to a range of 1–100 nm, and it covers a diverse application range in almost every field [1,2,3,4]. It is concerned with using particles by governing their size and shape at a scale of a billionth of a meter [1,4]. A much simpler interpretation is the generation of 100 nm or lower size functional structures [5,6]. Materials might indicate dissimilar characteristics at nanoscale dimensions; some might be better conductors of heat or electricity, some might have better stability, while the others possess distinct magnetic properties or better-reflecting light or color-changing properties in relation to their size [3]. Nanomaterials have much higher specific surface areas, making them ideal for a wide application range requiring large surface areas, including biocatalysis, molecular interactions of biomolecules in drug-delivery systems or energy-related applications.

The fast growth of nano-based technologies has led to the widespread utilization of nanomaterials in a wide variety of fields related to the chemical industry, materials science, electronics, aerospace industry, energy, environment, food, mechanics, robotics, quantum mechanics, protein engineering, tissue engineering, photonics, textile, cosmetics, sporting goods, including also medicine, biomedicine, and dental applications [7,8,9,10,11]. Nanotechnology has been regarded as the state-of-the-art technology of the recent era. According to the European Commission’s recommendation, the concept and the definition of nanomaterial have been revealed in relation to the material’s origin, formation, form, particle size, and size distribution [9,12,13].

### 1.1. History and Progress of Nanotechnology

The conception of nanotechnology was originally introduced by Feynman [14], who has been regarded as the ancestor of modern nanotechnology [12]. Taniguchi et al. [15] defined nanotechnology as the treatment of materials consisting of consolidation, separation and deformation caused by an atom or a molecule [4]. The famous form of carbon, which would find widespread applications in the following years called the carbon nanotubes, was for the first time introduced by Iijima in 1991 [12,16,17]. Bayda et al. [4] presented a comprehensive review on the historical development, progress, chemical, physical, and nanomedical applications of nanosciences. The methods developed for nanostructures can be considered as top–down and bottom–up methods. There are differences between these two methods in terms of rapidity of the process, product quality, and expenses. In the bottom–up approach, the nanostructures are built from the bottom via the manipulation of self-assembly of atoms/molecules in nanoscale dimensions in a controlled manner by chemical and physical methods. The top–down method refers to the decomposition of bulk materials into nanostructured particles by the employment of recently developed techniques such as lithography or precision engineering [4,18].

Drexler [19] interpreted the building up of complex machines from singular atoms that can independently regulate atoms and molecules producing self-assembly nanostructured particles, hence contributing to the development of molecular engineering theory. Drexler et al. [20] employed nanobots or assemblers concepts for nano-based processes in the medicinal field, which led to the usage of the nanomedicine term [4].

Nanotechnology has shown rapid development due to its auspicious potential. The addition of nanoparticles (NPs) significantly promotes the enhancement of the physical and mechanical characteristics of a material [21]. Kumar and Kumbhat [22] described the unique properties of nanomaterials in their study, while Jeevanandam et al. [23] presented the historical development, sources, toxic properties and regulations related to NPs and nanostructured materials. They discussed the classifications and sources of NPs along with nanostructured materials and their toxicological influences over cells and tissue.

**Figure 1:**
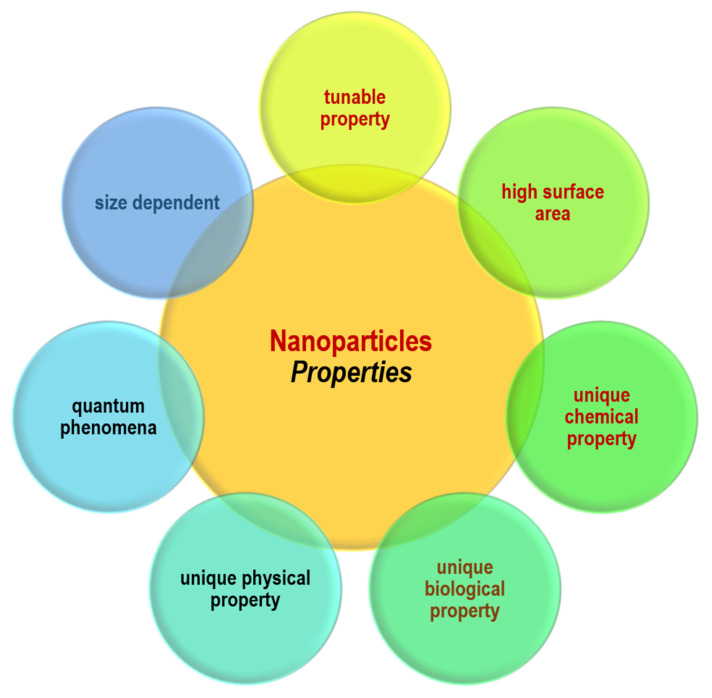
Unique properties of nanoparticles.

In the past few years, nanostructured materials have captured increasing attention owing to their promising applications in various fields involving diagnostics and therapeutics in medicine. This potential results from their unique chemical, biological and physical characteristics associated with their high specific surface area along with the quantum phenomena of the NPs. They are in a similar size range to most of the biological molecules such as proteins and membrane receptors. The interactions between these biomolecules and the NPs can be controlled by the proper adjustment of the surface property and the composition of the particles [9]. Figure 1 illustrates the unique properties of nanomaterials. Their behavior is significantly different from bulk materials due to the lower stability of atoms available on the surface. This is due to the fewer neighbors of the surface atoms, which will lead to lower coordination and unsatisfied bonds. The other reason is the quantum confinement effects due to the delocalization of electrons [9,24,25].

### 1.2. Nano-Dentistry and Contribution of Nanotechnology to Dental Applications

In the past few years, nanotechnology principles have contributed to the medical field for the improvement of treatment procedures [21]. NPs can be utilized efficiently as imaging, diagnostic and therapeutic agents [9]. Primary oral care and clinical dental sciences will continue experiencing considerable developments and progress at various levels of dentistry, which are of considerable benefits to both patients and dentists owing to the significant developments in the chemical, biological and physical sciences [6].

The nano-dentistry term was invented by Freitas [26], who contributed to developing nanorobots and nanomaterials. He conducted work on the regeneration of dentition and the development of dentifrobots in dentrifices, which are widely recognized by clinicians today [12,26,27]. Nanotechnology has contributed to the dental sciences with a diverse range of novel biomaterials, dental tissue regeneration templates, oral fluid nanodiagnostics, and lost dental hard tissue replacement NPs [6].

The most influential contribution of nano-based technologies to the field of conservative dental sciences is the utilization of various nanocomposites for the more improved restoration of tooth structure. In the near future, most of the procedures in dentistry are expected to be performed using types of equipment based on nanotechnology [28]. Markan et al. [28] presented a review on the progress of nanotechnological applications in endodontics, preventive and conservative dental sciences. In nano-dentistry, the object is maintaining ideal oral health by the use of nanomaterials, nanorobotics and tissue engineering [29]. Nanomaterials which are manufactured by either top–down or bottom–up approaches provide a novel insight in preventing and treating dental diseases [28,30,31]. The positively charged ions and the improved specific surface area enable the interaction of NPs with the bacterial cells carrying a negative charge, thus leading to a much higher antibacterial activity [32]. Moreover, NPs in the form of combined polymers or coatings onto biomaterial surfaces exhibit improved antimicrobial property [12,33].

### 1.3. Nanomaterial Characterization

The action and functioning of the NPs within their *in vivo* environment are greatly influenced by their physicochemical characteristics. NPs, in the biological medium, will soon encounter blood that would lead to agglomeration and segregation [34]. The optimum particle size for NPs is the 10–100 nm range for *in vivo* delivery, considering that the tiny particles are quickly sequestered by the reticuloendothelial system (RES) [35]. While NPs can assist as being beneficial diagnostic, imaging and therapeutic agents, they might cause some cytotoxic consequences. Therefore, the detailed characterization of NPs, especially for those designed for medicinal applications, is necessary to predict their behavior in biological systems [9]. 

The important current techniques that are useful for the characterization of nanomaterials have been described in various publications [9,36,37]. Surface characteristics, along with the particle size, is a significant factor for determining the life period and destruction of NPs during circulation. A hydrophilic surface of NPs would be favorable for the prevention of their interactions with the plasma proteins and inhibiting capture by RES [38,39]. It is necessary to predict and highlight the potential side effects along with their planned influence of NPs upon cell and tissues. Hence, the characterization and impact of NPs are crucial issues in their analysis [9].

Many techniques can be used for the structural, size and quantitative characterization of nanomaterials. Structural analysis techniques involve Fourier transform infrared (FTIR) spectroscopy, nuclear magnetic resonance (NMR), X-ray crystallography (XRD), Raman spectroscopy (RS), circular dichroism (CD) spectroscopy, site-directed spin labeling (SDSL), electron paramagnetic resonance (EPR), mass spectrometry (MS), dynamic light scattering (DLS), time of flight-secondary ion mass spectrometry (ToF-SIMS), fluorescence spectroscopy (FS), surface plasmon resonance sensor (SPR), fluorescence (FL) techniques, isothermal microcalorimetry (ITC), ultraviolet-visible spectroscopy (UV-VIS), and zeta potential (ZP). The size of NPs can be analyzed using techniques such as differential centrifugal sedimentation (DCS), ultracentrifugation (UC), asymmetrical flow field fractionation (AF4), differential light scattering (DLS), small-angle X-ray scattering (SAXS), scanning electron microscopy (SEM), small-angle neutron scattering (SANS), transmission electron microscopy (TEM), field-flow fractionation (FFF), sedimentation–velocity analytical ultracentrifugation (SV-AUC), and 2D analytical ultracentrifugation (2D AUC). Quantitative techniques involve MS, inductively coupled plasma-MS instrument (ICP-MS), MS-coupled with chemical, crosslinking (XL-MS), X-ray photoelectron spectroscopy (XPS), liquid chromatography-MS (LC-MS), fluorescence correlation spectroscopy (FCS), and fluorescence spectroscopy/tryptophan fluorescence (FS-TRP) [9,36,37].

### 1.4. Motivations and Aim of the Present Contribution

As pointed out, nanomaterials have gained importance lately in various fields owing to their unique properties, which yielded much improved performances. The impact of NPs in dentistry, as well as endodontics for the treatment of various dental infections, is showing fast progress. Studies have shown that NPs could serve as useful agents for many significant functions in endodontics, achieving beneficial results and having shown great promise in the field. Nanotechnology is expected to impact the diagnosis and materials in dental applications [40]. The results have been inspiring enough for more clinical investigations, which will enable the therapeutical importance of nano-structural particles to be verified. On the other hand, the literature studies have shown that the significance of nanotechnological improvements on endodontics is yet to be recognized. There is a need to highlight the progress and improvements in endodontic applications, such as the type of novel nanomaterials that would be prepared for the further progression of endodontic therapeutic agents; nano-testing methods that would enable the more precise detection and evaluation of diseases with choosing the most applicable therapeutics, and the role of NPs in imaging, diagnostics and therapeutics, in relation to the physicochemical and biological properties of NPs characterization. In addition, most studies are related with NPs’ *in vitro* environment, and a significant knowledge gap exists regarding predicting their behavior within an *in vivo* environment. Hence, there is a need to further address the detailed characteristics of NPs intended for medical applications, challenges in relation to the cytotoxic effects, and the issues related with the future development of nano-approaches in endodontics. In this context, this study aims to review the current technological progress and recent research outcomes as well as possible prospective applications of nanomaterials and nanotechnology in the area of endodontics.

In this study, recent progress and studies on nano-dentistry applications in endodontics have been investigated and presented in two parts. In the first part, nanoapplications including nanomaterials or the nano-improvement of materials in endodontics are discussed, while the second part involved the nanoapplications related to nano-testing of structures.

## 2. Materials and Methods

In the existing study, an extensive literature survey is carried out on the utilization of nanotechnology in endodontics, and the current status and applications of nanotechnology in endodontics are discussed with illustrative examples. On that account, a manual electronic survey was carried out through the electronic databases such as Web of Science, Google Scholar, and PubMed, using keywords “nano-dentistry”, “dental nanomaterials + endodontics”, “nanotechnology + endodontics”, “nano-identification techniques + endodontics”, “nano-characterization techniques + endodontics”. Relevant articles published between 2008 and 2022 were retrieved, and a preliminary analysis was performed among the publications by screening the titles and abstracts of the published articles. The inclusion criteria for the search involved the original data available including original articles published in English at peer-reviewed indexed journals. The exclusion criteria were non-English studies, letters, and articles not involving data. Full texts of the articles were retrieved, then with an additional manual search, we added publications on the topic on recent studies including literature reviews. A comprehensive literature survey was achieved to determine the current and possible future applications of nanotechnology in endodontic treatments. The methods that are useful for the characterization of nanomaterials as well as recent applications of nanomaterials in endodontics, nano-testing of dental structures in endodontic instruments, conclusions and challenges in the field on the selected representative articles are described.

## 3. Applications for Nanotechnology in Endodontics

Nanotechnology has been utilized in several different parts of dentistry [41]. Nanomaterial studies have resulted in the development of novel materials which improved the clinical outcomes significantly [6]. The scope and applications of nanotechnology in endodontics have been described in a number of studies in the literature [2,6,12,21,28,41].

Successful endodontic treatment involves some major procedures such as biomechanical steps, disinfection, 3D sealing, and obturation of the root canal system [42,43]. Failure might occur owing to the insufficiency of biomechanical steps related with the root canal system anatomy, also the microleakage of sealing materials, in spite of the many successful root canal treatment applications. The microleakage arises mostly due to the probable deficiencies in the quality [44,45]. The irrigant activation and cleaning plays an important role in the success and failure of endodontic treatment. It was reported that irrigant activation promoted better pulp tissue dissolution in comparison to syringe/needle protocol, and pulp tissue dissolution was significantly higher when heating was followed by sonic/ultrasonic activation [46,47]. Some materials used in endodontics might have specific shortcomings such as shrinkage, moisture sensitivity, and dissolution in the oral medium [48]. Progress in the synthesis of novel materials having better quality sealing and biomechanical features will enable the endurance of the success of endodontic treatment [6].

Research related to the nano-applications in endodontics has been inaugurated in several treatment fields. Nanomaterials having superior resistance toward wear and fatigue are proposed for the surface modification of rotary nickel–titanium (Ni-Ti) files presently used in root canal treatment. NPs would be effective for the improvement of the medicament, irrigant, sealers, and obturating materials, and drug elutions would help for the improvement of sealing and disinfection of root canal systems. Regenerative endodontics applications are currently in progress, generating enhanced scaffolds [41].

There are a considerable amount of successful research activities progressing in the field of endodontics which attempt to amend several clinical directions such as files and filling constituents. Some NPs show better antimicrobial characteristics, which can improve the efficacy of endodontic materials, intracanal medicaments and irrigation solutions, because of their capability to spread better into the complex anatomical parts of the root canal systems owing to their particle size [6,49,50]. Most studies have continuously concentrated on the synthesis and utilization of nanocomposites obtained by the modification of nanomaterials. These NPs could fortify the sealing characteristics during obturation, which could be applied as root repair and root-end filling materials [6,50,51,52].

### 3.1. Nanomaterials in Endodontics

**Figure 2:**
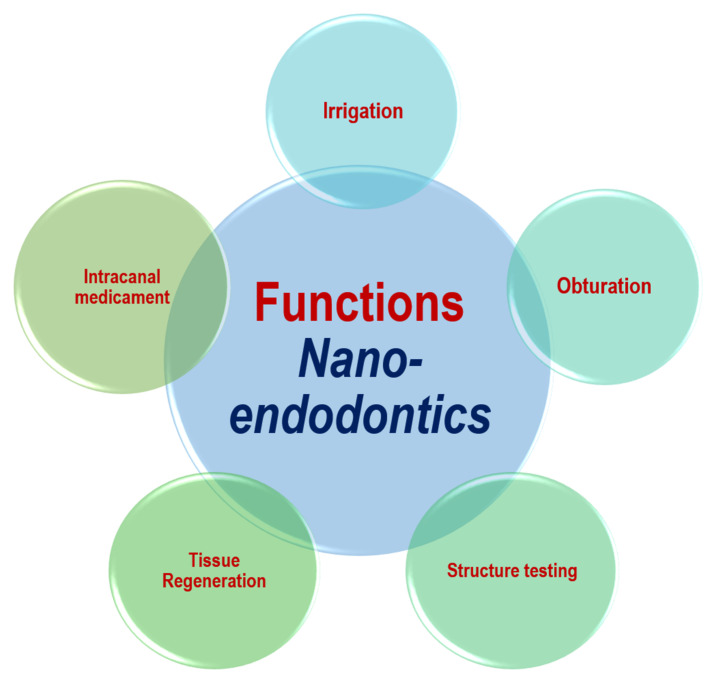
Functional applications of nanotechnology in endodontics.

**Figure 3:**
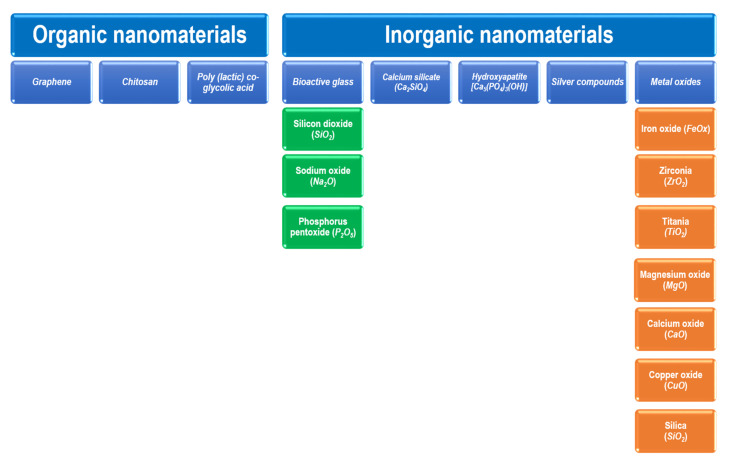
Types of nanomaterials employed in endodontic applications.

Nanomaterials can be employed as irrigation, intracanal medicament, obturation materials and sealers [12]. The functional applications of nanomaterials in endodontics are shown in Figure 2. The types of nanomaterials in endodontic applications can be mainly classified as organic NPs and inorganic NPs. Graphene, chitosan, and poly (lactic) co-glycolic acid are among the organic nanomaterials that are used. Inorganic nanomaterials involve bioactive glass, calcium silicate (*Ca*_2_*SiO*_4_ or *2CaO·SiO*_2_), hydroxyapatite (*Ca*_5_(*PO*_4_)_3_(*OH*)), silver compounds, and metal oxides. Bioactive glass can be composed of silicon dioxide (*SiO*_2_), sodium oxide (*Na*_2_*O*), and phosphorus pentoxide (*P*_2_*O*_5_). Metal oxides may involve the oxides of iron (*FeOx*), zirconium (*ZrO*_2_), titanium (*TiO*_2_), calcium (*CaO*), magnesium (*MgO*), copper (*CuO*), and silicon (*SiO*_2_). Such nanomaterials have shown promising results in endodontics [12,53]. Figure 3 shows the main types of nanomaterials employed in endodontic applications. However, it should be noted that the materials might somehow be subject to long-term degradation. Thus, the issues related with the problems of deterioration that might occur simultaneously need to be taken into consideration. In addition, the problems of aging or long-term degradation due to either radiation degradation manifesting during radiation treatments or surface degradation might also be significant [54,55].

It has been confirmed that the most efficient disinfection of root canals could be through the use of NPs owing to their broad-spectrum antibacterial activities [2,53,56]. The nanomaterials evaluated for disinfection in endodontics practice include zinc oxide, silver chloride and chitosan nanoparticles [53]. The efficacy of zinc oxide and chitosan against *Enterococcus faecalis* (*E. faecalis*) was owing to their capability to break up the cell wall. Additionally, these have the ability to break down the biofilms within the root canals [56,57,58]. Silver NPs are effective as root canal disinfection agents. It was indicated that a silver NP (0.02%) gel was able to disrupt and kill the *E. faecalis* biofilm [59,60].

A kind of bioactive glass NPs have been employed efficiently as an antimicrobial agent for root canal disinfection [61,62]. Its antimicrobial effect is owing to its capability to sustain the alkaline environment for a longer time. Studies have shown that an increase in silica release and pH level was possible via the employment of a specific type of a nanometric bioactive suspension and micrometric hybrid material [28,63]. Fioretti et al. [64] reported the use of multilayered functional groups involving nanostructured films which contain a melanocortin peptide as a novel biomaterial for endodontic regeneration.

Recent progress and some selected studies that investigated applications of nanomaterials in endodontics are presented in Table 1. The topic of the study, the type of nanomaterials used, and the methods followed related with each specific study are indicated with the corresponding references. Table 2 presents the results and achievements on the application of nanomaterials in endodontics with respect to the targeted objectives of each study.

Mineral trioxide aggregate (MTA) has been utilized commonly in most endodontics applications. Some studies concerned the nano-modification of this material. For example, Saghiri et al. [65] investigated the nano-modification of MTA and investigated the enhancement of its physiochemical properties. They analyzed the properties of WMTA (white) and NWMTA (nano white) and made a comparison. After preparation, WMTA and NWMTA were mixed together. The specific surface area before hydration, setting time, XRD and microhardness at different pH conditions were evaluated by BET, ISO 6876, Vickers hardness and EDS analysis techniques, respectively, for both materials. Significant differences in specific surface area, surface hardness and setting time were observed for the two materials. It was reported that an increase in specific surface area resulted in reduced setting time and increased microhardness even at lower pH conditions [65]. In another study, the changes of material physical properties and setting time were studied by Akbari et al. [66] when nano-SiO_2_ was added to WMTA. It was observed that nano-SiO_2_, which acted as a filler in cement, improved the microstructure and accelerated the process of hydration. The influence of a nano-BG (58S) for the odontogenic differentiation and mineralization of human dental pulp cells (hDPCs) was investigated by Gong et al. [67] using an *in vitro* analysis. Their study involved the preparation of extractions from the incubation of various particulates (58S, 45S5, nano-58S BG) in Dulbecco modified Eagle medium. They used the BG extractions in which the hDPCs were cultured as supernatants, and they studied the proliferation of hDPCs and the odontogenic differentiation depending on the polymerase chain reaction of genes related with differentiation and mineralization (ALP, collagen type I, DSPP, dentin matrix protein 1). They examined the gene expressions through ALP activity evaluation, osteocalcin and DSPP immunocytochemistry staining, and mineralization assay. They reported that the nano-bioactive glass induced the differentiation and mineralization of hDPCs more effectively, and it could be proposed as a potential candidate for the regeneration of the dentin–pulp complex [67].

Saghiri et al. [68] evaluated the impact of bismuth oxide as a radiopaque additive, investigating the influence of particle size and radiopacity of some type of cements based on tricalcium silicate (CSC). Different CSC kinds were used in the study (CSC, CSC + 10% bismuth oxide (10 μm), CSC + 20% bismuth oxide, CSC + 10% nano-bismuth oxide (50–80 nm), CSC + 20% nano-bismuth oxide, nano-WMTA (40–100 nm)). The radiopacity, compressive strength and surface microhardness analysis were carried out on the samples. It was reported that the 20% nano-bismuth oxide addition improved the physical properties without any considerable change of radiopacity, and lower values of physical properties were observed with the 10 μm sized bismuth oxide-containing CSC material. In another study, the biocompatibility of the NPs based on active CS and hydroxyapatite (HA-CS) systems was studied by Petrović et al. [69], in which the *in vitro* cytotoxic and *in vivo* inflammatory responses to the materials were evaluated. In the followed methodology, the cytotoxicity of the eluates of the materials was examined employing the MTT assay on MRC-5 cells. Test samples involving polyethylene tubes were implanted in the subcutaneous tissue of Wistar rats, and histopathological evaluation was carried out. According to the results, HA-CS caused rather thick capsules, while MTA (control) resulted in thin capsule formation. Cytotoxic and inflammatory response evaluations showed the more effective biocompatibility of CS and HA-CS as compared to MTA [69]. According to the literature reports, the outcome of HA NPs indeed depends on their resistance to aging, including radiation [54,55].

The evaluation of the angiogenic properties of some vital pulp therapy materials such as WMTA, calcium hydroxide (CH), Geristore, and nano-WMTA was investigated in a study conducted by Saghiri et al. [70]. In the study, materials were prepared in the form of disks dispersed into water; then, they were centrifuged for obtaining supernatant elution. Mice molar endothelial cells (ECs) were left on the prepared hydrogel arrays. For the evaluation of the investigations according to the choroidal neovascularization (CNV) model, female mice (6 weeks old) were laser treated, and elution from samples were taken by injection on the laser running day and after 1 week. It was reported that the results indicated minimum antiangiogenic activity, while Geristore and nano-WMTA confirmed more significant proangiogenic activities [70].

Naseri et al. [71] evaluated the microhardness and superficial chemical structure of radicular dentin by the addition of nano-CH by an *in vitro* work. It was reported that CH and NCH were effective in intracanal medicaments on the chemical and physical features of dentin. In the trial, a number of dentin discs were prepared as control and treatment groups using pastes of CH and NCH. Dentin microhardness was assessed after a certain period of time using the Vickers test, and the FTIR analysis was used for chemical characterization. The use of CH in intracanal medicament reduced the microhardness of dentin (4 weeks), while NCH did not cause any change; on the other hand, a chemical structure change was observed 1 week later for both materials [71].

In a study, Yang et al. [72] investigated the antibiofilm effect of an auxiliary irrigant solution (proanthocyanidin PA) on *E. faecalis* along with the effects on the biodegradation and mechanical resistance properties of demineralized root dentine. In their followed method, *E. faecalis* was added into human root dentine tubules applying a series of centrifugation procedures and then left to grow for a period of 1 week. Dentine blocks affected by *E. faecalis* were processed with various irrigant solutions such as sterile water, chlorhexidine (CHX), and PA. Then, the bacteria within *E. faecalis* biofilms (live, dead) were identified using a confocal laser scanning microscope. The hydroxyproline release and elastic modulus of human dentine were characterized for the evaluation of the biostability. The demineralized dentine collagen was analyzed by XPS for surface chemical characterization. According to the results, PA was effective at killing *E. faecalis* within biofilms and improved the biostability of the demineralized root dentine collagen matrix. It was proposed that PA could be applied effectively as an auxiliary endodontic irrigant for antibiofilm and collagen stabilizing [72]. In another study, the regeneration of copper–calcium hydroxide (Cupral)-endodontically treated teeth with apical periodontitis was evaluated via the employment of an electrophoresis technique by Meto et al. [73]. It was reported that the Cupral-electrophoresis methodology was effective in treating destructive periodontitis of teeth with problematic canals up to 18 months allowing teeth preservation.

### 3.2. Nano-Testing of Structures in Endodontic Applications

The Ni-Ti endodontic rotary file is one of the widely utilized instruments in dental applications. The alloys employed have many advantages such as high resistance to corrosion and superelasticity that grant them with good shape memory. So, exploring the complicated anatomy of the root canal for a suitable endodontic treatment would be possible. It was reported that cobalt coatings of a Ni-Ti file with fullerene-like WS2 impregnation on NPs resulted in considerable improvements in the breakage time and fatigue resistance [6].

External cervical resorption (ECR), which is the dental hard tissue loss due to the action of odontoclasts, associates dental, periodontal and pulpal tissues in the following phases and involves a dynamic mechanism. Over the recent years, ECR has drawn increased attention, owing to the advanced micro-CT, histopathological and radiographic CBCT detection techniques. However, it is reported that further work is necessary for the confirmation of the causes and effects of some possible influencing factors. The maxillary central incisor, maxillary canine, maxillary lateral incisor, mandibular first molar and maxillary first molar teeth are mostly affected, respectively. The corresponding steps in the ECR process involve initiation, following progression and resorption, then reparative phases. Resorption, repair or remodeling might develop concurrently at varying parts of the infected tooth. The improved accuracy of CBCT analysis leads to the more accurate identification and evaluation of ECR along with determination of the best treatment procedure [74].

Recent progress and some selected studies investigated on applications of nano-testing methods in endodontics are presented in Table 3. The topic of the study, the type of nano-testing method employed, and the procedures related with each specific study are indicated in the table with the references. Table 4 shows the results and achievements on the application of nano-testing methods in endodontics with respect to the targeted aims of each study.

In a study conducted by Zinelis et al. [75], a nano-indentation technique was used for the evaluation of the in-depth hardness profiles of Ni-Ti and stainless steel (SS) instrument cross-sections, utilizing three instruments of each kind. Hardness profiles were determined toward the center (2000 nm) by an MTS XP nanoindenter. According to the results, a drop in hardness was observed as moving to the center for the endodontic instruments, which implied that the surface hardness was considerably improved depending on the applied techniques. The cyclic fatigue effect on Ni-Ti endodontic instruments was investigated by Jamleh et al. [76] using a nano-indentation analysis. In the investigation, several numbers of fractured and new Ni-Ti rotary instruments were analyzed, and they reported that the technique could be utilized for the determination of the performance along with the failure mechanism of the instruments. The fatigue analysis indicated a considerable decrease in the elastic modulus and hardness of the instruments.

A methodology composed of a combination of techniques for the investigation of a central incisor with a large ECR case was introduced in a study by Mavridou et al. [77]. The diagnosis was based on clinical inspection, cone-beam computed tomography (CBCT) and digital radiography. The tooth was examined by micro-CT, nano-CT and hard tissue histology after extraction. It was reported that the nano-CT was more efficient than the other techniques. The reparative tissue, pulp tissue reactions, pericanalar resorption-resistant sheet, resorbed canals and their interconnection with the periodontal ligament space were determined by nano-CT technique. The methodology was proposed as a fast and minimal invasive method to study the ex vivo evaluation of ECR together with the hard tissue histology. The approach of combining clinical and CBCT along with the nano-CT and histological mapping analysis was proposed as an ideal method for ECR identification. Mavridou et al. [78] studied the mechanisms and properties of ECR patterns in endodontically treated teeth and teeth with vital pulps (seven cases of each). The diagnosis depended on clinical and radiographic CBCT analysis results. The extracted teeth were also examined by SEM, hard-tissue histology and nano-CT. They observed similar ECR patterns consisting of initiation, resorption, and then reparative steps in all the teeth, while some differences were observed in the resorption and reparative stages between the two types of teeth. The resorption stage in root canal-filled teeth was more intense than the repair stage due to the clastic cells and the presence of granulation tissues as well as the absence of the pulp and protective PRRS layer and due to the chemical composition change of the root dentine after root canal treatment [78].

A study was conducted by Iacono et al. [79] for the comparison of the phase transformation behavior, microstructure, nano-hardness, elasticity modulus and the surface chemistry of HyFlex EDM and conventional HyFlex CM instruments, using analysis techniques such as XRD, DSC, Raman spectroscopy and FE-SEM. Considerable differences in measurement results of elasticity modulus and nano-hardness were determined between the two files. HyFlex EDM revealed enhanced phase transformation temperatures and hardness. Analysis results confirmed the enhanced mechanical behavior of the instruments [79].

A study was carried out by Petitjean et al. [80] on the assessment of a calcified extraradicular deposit on the apical root surfaces of both roots by the application of a multimodular approach involving a combination of multiple investigation methods. The root contained an apical periodontitis lesion, and a sinus tract was the only connection with the oral cavity. The related diagnosis and treatment were achieved using clinical, ultrasound and radiographic (2, 3D) examinations. Microscopic imaging, electron probe microanalysis (EPMA), hard/soft tissue histology and nano-CT were also used for the analysis of the structure and composition of the extraradicular deposit [80]. In a review paper presented by Patel et al. [74] on the histopathology and distribution of ECR, it was emphasized that CBCT appeared as a better technique because of the limited performance of regular periapical radiography in the detection and assessment of ECR.

Contrast-enhanced nano-CT was used by Hildebrand et al. [81] to investigate the dental ultrastructures (soft dental tissues, cellular layers) utilizing phosphotungstic acid (PTA) as an agent. In the method, sound third molars from healthy adults were put in paraformaldehyde buffered solutions, and the influence of PTA concentration on dental hard and soft tissues for CT identification was evaluated. The samples were also analyzed using a high-resolution nano-CT for the examination of the cementum and pulpal sections. A 3D investigation along with quantitative analysis of the dentine composition was obtained via the segmentation of the sigmoidal dentinal tubules and the surrounding dentine. It was reported that the staining protocol allowed the visualization of hard and soft tissues along with cellular layers in teeth using nano-CT imaging, and the protocol depended on the tissue type and size. The method offered an improved opportunity for the concomitant visualization of hard and soft dental tissues [81].

In a study, nano-CT analysis was utilized in the evaluation of the total obturation volumes and voids for various obturation techniques by Holmes et al. [82]. The study was based on the consideration that the material and the technique used did not have any effect on the total obturation volume or voids. Using maxillary left central incisor 3D-printed replicas and different obturation groups in the investigation, nano-CT along with volumetric analysis were performed after obturation. It was reported that the materials and the obturation technique considerably affected the voids and the total volume of obturation material [82]. The disinfecting and shaping characteristics of some preparation protocols in C-shaped root canals were evaluated by Gazzaneo et al. [83] employing a correlative microcomputed tomographic and molecular microbiology work. The BioRaCe and XP-endo Shaper systems were reported to have similar disinfection and shaping properties in mandibular molars having C-shaped canals. In addition, the supplementary steps with the Hedström file and the XP-endo Finisher promoted similar decreases in the unprepared canal surfaces, whereas the effects were not sufficient to have significant improvements in bacterial elimination; thus, the development of more effective strategies would be needed for the disinfection of the mentioned canals [83].

## 4. Discussion of Literature Work

The impact of the development of novel NPs, their surface modifications, enhanced biofunctional and tribomechanical behaviors, clinical performances, along with various nano-characterization methodologies in the dental field involving endodontics has been emphasized in a number of recent contributions as well [84,85,86,87,88,89,90,91]. Studies have shown that the impact of nanomaterials for the treatment of various oral diseases in endodontics is showing fast progress. Nanomaterials can perform as beneficial imaging, diagnostic and therapeutic agents [9,12]. They can be employed as fillers, irrigants and photodynamic therapy for the achievement of useful results [21]. NPs have shown considerable potential in the reduction in biofilm formation, remineralization enhancement of the tooth structure by the inhibition of demineralization and counteract of caries and endodontic microorganisms. The results have been inspirational for further clinical research for nano-based materials to be authenticated [12]. Antibacterial NPs can be employed for disinfection and have indicated better efficacy in avoiding bacterial cells. Additionally, nanomaterials can be applied to sealers required in endodontics. Nanomaterials can enhance the anti-leakage property of the sealer. Nanotechnology can also be applied in photodynamic therapy in endodontics, with possible enhancements in the efficacy of the method [21].

The literature studies have shown that the significance and consequences of nanotechnology on endodontics applications still need to be appreciated. Nanoapplications are appropriate to root canal therapy in many aspects, such as canal irrigation, obturation, instrumentation, canal sealers, endodontic regeneration, and pulp repair. The improvements and applications are encouraging for sustainable novel materials for clinical dental applications [6].

The employment of nanocomposites can accelerate the hydration process, thus reducing the setting time without any significant influence on the flexural and compressive strength of materials. Furthermore, adding nanomaterials to regular dental materials and the formation of nanocomposites enhance the physical features of materials, with no remarkable radiopacity changes. An increased specific surface area can lower setting time and increase microhardness. Nano-BG materials can induce the differentiation and mineralization of human dental pulp cells more efficiently as compared to regular ones. Nano WMTA show better proangiogenic activity. Proanthocyanidin was effective in killing *E. faecalis* within biofilms and in enhancing the biostability of the collagen matrix of demineralized root dentine. Its usage as an auxiliary endodontic irrigant with collagen-stabilizing and antibiofilm effects was recommended [65,66,67,68,70,71,72]. Nanomaterials were proposed as potential candidates for the regeneration of dentin–pulp complexes.

The nano-indentation technique can be employed for the determination of the performance of Ni-Ti devices [76]. Nano-CT has been shown to be a rapid and minimal invasive technique for the analysis of ECR ex vivo, which can be complementarily employed with hard tissue histology. Clinical and CBCT analysis, nano-CT along with histological mapping are ideal methods for ECR identification [77]. The materials and the obturation techniques considerably affect the voids and the total volume of obturation material [81]. The use of HyFlex EDM revealed the enhanced mechanical behavior of these instruments [79]. The progress and improved accuracy of CBT techniques led to the better identification, evaluation and selection of the most appropriate treatment plans for ECR [74]. High-resolution CBCT, nano-CT, intra-oral radiography, hard tissue histology and electron probe microanalysis can enable better characterization of the extraradicular deposit formation [80]. By following a staining protocol which depends on tissue type and size, the detection of hard and soft tissues along with cellular layers in teeth could be possible, making use of a nano-CT technique. Enhanced possibilities for the concomitant visualization of soft and hard dental tissues would be possible [81].

## 5. Conclusions and Outlook

Nanotechnology has introduced many novel materials and techniques in the dental field involving the endodontics. NPs can be effectively used in endodontic applications, while the physicochemical and biological properties have to be well characterized. Studies have shown that the significance and consequences of nanotechnology on endodontics applications still need to be appreciated. NPs, with their great specific surface areas, might be sensitive to contamination during processing conditions. The behavior of NPs *in vivo* is rather different than in an *in vitro* environment. The cytotoxicity of NPs *in vivo* needs to be explored. Consequently, it is necessary to perform a detailed characterization of NPs, specifically those planned for medical applications for the prediction of their behavior in the *in vivo* environment. Although NPs have shown considerable potential in endodontic applications, there still exist some issues such as biocompatibility, human safety, ethical and economical-related problems that need to be addressed. Nanodentistry is a developing field with a strong potential by new techniques in the diagnosis and treatment of dental diseases; thus, the future of dentistry and endodontics is open to further progress and changes with developments in nanotechnology.

## Figures and Tables

**Table 1:** Nanotechnology applications in endodontics: nano-improvement of materials.

Study	Nanomaterials/Methods	Reference
nano-modification of MTA for enhanced physicochemical properties	preparing WMTA, nano-WMTA and mixing powder surface area (BET), setting time (ISO-6876), micro-hardness (Vickers), XRD measurements	Saghiri et al. [65]
effect of nano-silica on setting time and physical properties of MTA	adding nano-silica to WMTA, mixing with water setting time, compressive strength, flexural strength measurements comparing with pure MTA	Akbari et al. [66]
effect of nano-BG on differentiation and mineralization of hDPCs	investigating the effect of nano-BG (58S) on the odontogenic differentiation and mineralization of hDPCs in vitro. preparing BG (nano-58S, 45S5, 58S) extractions by incubation in Dulbecco modified Eagle medium (1% *w*/*v*, 24 h), filtrating (0.22 μm) culturing hDPCs in BG extractions evaluating proliferation of hDPCs using methylthiazol tetrazolium assay evaluating odontogenic differentiation and mineralization ALP activity assessment, immunocytochemistry staining, mineralization assay	Gong et al. [67]
effect of radiopacifier particle size on the physical properties of CSC	impact of nano-bismuth oxide on the physical properties and radiopacity of CSC preparing CSC types ▪ *CSC* ▪ *CSC + 10% bismuth oxide* (*10* μm) ▪ *CSC + 20% bismuth oxide* ▪ *CSC + 10% nano-bismuth oxide* (*50–80* nm) ▪ *CSC + 20% nano-bismuth oxide* ▪ *nano WMTA* (*40–100* nm) surface microhardness, radiopacity, compressive strength tests	Saghiri et al. [68]
biocompatibility of nanomaterials: *in vitro* and *in vivo* study	nanomaterials (CS, HA-CS, MTA) testing cytotoxicity of nanomaterial eluates using MTT assay on MRC-5 cells implanting test materials in subcutaneous tissue of Wistar rats histopathological examinations	Petrović et al. [69]
hydrogel arrays and CNV model for angiogenic activity of vital pulp therapy biomaterials	angiogenic properties of vital pulp therapy materials (WMTA, CH, Geristore, nano-WMTA) preparation of material (WMTA, CH, Geristore, nano-WMTA) disks, obtain supernatant elution. preparation wells of polyethylene glycol hydrogel arrays, placing mice molar ECs sample elutions added to hydrogel arrays 6-week-old female mice (35) lasered, elution from each sample/saline delivered by intravitreal injection on laser treatment day and after 1 week CNV model evaluation	Saghiri et al. [70]
effect of nano–CH on microhardness and superficial chemical structure of root canal dentin: ex *vivo* study	effect NCH on the microhardness and superficial chemical structure of radicular dentin, in vitro trial 80 dentin discs assigned into control and treatment groups CH and NCH pastes used in groups samples were washed with saline/sodium hypochlorite after 1 and 4 weeks, Vickers test for dentin microhardness, FTIR for phosphate/amide I	Naseri et al. [71]
antibiofilm and collagen-stabilizing effects of PA as an auxiliary endodontic irrigant	introducing *E. faecalis* into human root dentine tubules by centrifugation treating dentine blocks infected with 1-week-old *E. faecalis* biofilms with irrigants (sterile water, CHX, PA). analyzing bacteria (live/dead) within *E. faecalis* biofilms (confocal laser-scanning microscopy) evaluating biostability of fully demineralized dentine treated by irrigants testing elastic modulus and hydroxyproline release of human dentine incubated in collagenase solution (at baseline, after irrigant treatment, biodegradation) XPS characterization of demineralized dentine collagen treated by irrigants	Yang et al. [72]

**Table 2:** Achievements related to nanotechnology applications in endodontics: nano-improvement of materials.

Aim	Results and Achievements	Reference
analyzing physicochemical properties of nano-NWMTA	increasing surface area of powder can reduce setting time and increase microhardness even at lower pH values after hydration	Saghiri et al. [65]
evaluating the effect of nano-silica to MTA on setting time and physical properties	addition of nano-silica to MTA accelerated the hydration process, reduced the setting time, and had no adverse effect on the compressive and flexural strength of MTA	Akbari et al. [66]
investigating the effects of nano-BG on hDPCs *in vitro*.	Nano-BG (58S) can induce the differentiation and mineralization of hDPCs more efficiently, potential candidate for hDPCs regeneration	Gong et al. [67]
evaluating effect of nano-bismuth oxide as radiopaque additive, particle size on the physical properties, and radiopacity of CSC	addition of 20% nano-bismuth oxide enhanced physical properties of CSC, no significant radiopacity change regular particle-size bismuth oxide reduced the physical properties of CSC	Saghiri et al. [68]
evaluating in vitro cytotoxicity and in vivo inflammatory response to nanomaterials	materials significantly reduced cell viability CS, HA-CS significantly less toxic than MTA cytotoxicity could be partially attributed to pH kinetics over time dilution of eluates of materials resulted in better cell survival histopathological examination indicated similar inflammatory reaction, vascular congestion, connective tissue integrity (CS, HA-CS, MTA) HA-CS induced moderately thick capsules, MTA resulted in thin capsule formation better biocompatibility of CS, HA-CS as compared to MTA	Petrović et al. [69]
evaluating angiogenic properties of vital pulp therapy biomaterials, hydrogel arrays and CNV models	all materials showed minimal antiangiogenic activity Geristore and nano-WMTA showed higher proangiogenic activity	Saghiri et al. [70]
evaluating effect of nano–CH on microhardness and superficial chemical structure of radicular dentin	CH as an intracanal medicament for 4 weeks reduced dentin microhardness no change in microhardness with nano-CH change in the superficial chemical structure observed earlier after 1 week for both CH, nano-CH groups	Naseri et al. [71]
evaluating antibiofilm effect of PA solution as an irrigant against *E. faecalis* evaluating influence on mechanical properties, biodegradation resistance of demineralized root dentine	dead *E. faecalis* volume significantly higher in PA, CHX groups PA irrigation significantly increased mechanical properties of demineralized dentine, effect was enhanced with increasing PA concentration CHX, PA groups had significantly less elasticity loss and hydroxyproline release dentine collagen biomodification by PA verified by XPS PA killed *E. faecalis* within biofilms and enhanced biostability of the collagen matrix of demineralized root dentine. PA might be used as an auxiliary endodontic irrigant with antibiofilm and collagen-stabilizing effects	Yang et al. [72]

**Table 3:** Nanotechnology applications in endodontics: nano-testing of structures.

Study	Methods	Reference
in-depth hardness profiles of SS/Ni-Ti endodontic instrument cross-sections by nano-indentation	studying SS/Ni-Ti instruments measuring in-depth hardness profiles after embedding/metallographic preparation using MTS XP nanoindenter (Berkovich diamond)	Zinelis et al. [75]
nano-indentation testing of new and fractured Ni-Ti endodontic instruments	testing Ni-Ti rotary instruments using a cyclic fatigue set-up until fracture using fractured and new Ni-Ti instruments for nano-indentation test	Jamleh et al. [76]
a multimodular methodology for investigating ECR	selecting one case of a central incisor (extensive ECR) to demonstrate the potential of methodology ECR diagnosis based on clinical inspection/digital radiography/CBCT investigating tooth using micro-CT/nano-CT/hard- tissue histology (after extraction) comparison of techniques	Mavridou et al. [77]
understanding ECR patterns in endodontically treated teeth	investigating cases of endodontically treated permanent teeth displaying ECR ECR diagnosis based on clinical findings/radiographic examination with CBCT further analysis of extracted teeth by nano-CT/hard-tissue histology/SEM	Mavridou et al. [78]
structural analysis of HyFlex EDM instruments	examining new and laboratory used HyFlex EDM by XRD/DSC investigating nano-hardness/elasticity modulus using RS, FE-SEM to assess the surface chemistry of HyFlex EDM investigations with HyFlex CM for comparison	Iacono et al. [79]
multimodular assessment of a calcified extraradicular deposit on the root surfaces of a mandibular molar	a mandibular first molar with a calcified extraradicular deposit on the apical root surfaces of both roots an apical periodontitis lesion/a sinus tract serving as the only communication with the oral cavity diagnosis/treatment based on clinical/radiographic (2–3D)/ultrasound examination further analyzing the tooth using microscopic imaging, nano-CT, hard and soft tissue histology, electron probe microanalysis after extraction	Petitjean et al. [80]
ECR: histopathology, distribution, presentation	novel micro-CT, histopathological techniques, radiographic detection using CBCT review covering the etiology, potential predisposing factors, histopathology, diagnosis of ECR	Patel et al. [74]
contrast-enhanced nano-CT revealing soft dental tissues and cellular layers	collecting sound third molars from healthy human adults (buffered paraformaldehyde) evaluating impact of PTA on dental soft/hard tissues for CT imaging cementum/dentine-pulp sections cut, dehydrated, stained (12, 24 h; 2, 5 days) scanning samples by nano-CT to examine cementum/pulpal regions	Hildebrand et al. [81]
volumetric evaluation of root canal obturation methods in truetooth 3D-printed tooth replicas using nano-CT	evaluating the volumes of total obturation and voids in different obturation techniques using nano-CT imaging fifty maxillary left central incisor 3D-printed replicas (truetooth) instrumented, assigned to 5 different obturation groups: ▪ *single cone with EndoSequence Gutta-Percha Points and Ribbon Sealer* ▪ *single cone with BC 150 Series Gutta-Percha Points and EndoSequence BC Sealer* *continuous wave with EndoSequence Gutta-Percha Points and Ribbon Sealer* ▪ *GuttaCore carrier obturation and Ribbon Sealer* ▪ *cold lateral condensation with EndoSequence Gutta-Percha Points and Ribbon Sealer* after obturation, obtaining nano-CT images, performing volumetric analysis	Holmes et al. [82]

**Table 4:** Achievements related to nanotechnology applications in endodontics: nano-testing of structures.

Aim	Results and Achievements	Reference
evaluating in-depth hardness profiles of SS/Ni-Ti endodontic instrument cross-sections using a nano-indentation technique	for all instrument cross-sections, maximum hardness obtained at the outer surface hardness classification of instruments, for both outer and innermost locations, in decreasing order: Reamer > K > Hedström > Profile > NRT shank (without thermal treatment) > NRT tip (with thermal treatment) > Liberator maximal hardness, at the outer surface of instruments attributed to residual stresses developed due to cutting and thermal effects during manufacturing increased outer layer hardness may have a beneficial effect on cutting ability and wear resistance of instruments all endodontic instruments had a decrease in hardness toward their center, implying that the surface hardness of contemporary instruments was significantly enhanced by the consequences of manufacturing processes	Zinelis et al. [75]
investigating effect of cyclic fatigue on Ni-Ti endodontic instruments using a nano-indentation test	significant differences in terms of hardness/elastic modulus for groups nano-indentation technique can be applied to determine the performance/failure mechanism of Ni-Ti instruments fatigue process revealed a significant decrease in hardness/elastic modulus of Ni-Ti instrument fatigue process did not result in work hardening but rather work softening	Jamleh et al. [76]
introducing a multimodular combination of techniques as a novel minimal invasive approach efficient and accurate investigation of ECR	nano-CT is a fast and minimal invasive technique for ex vivo analysis and understanding ECR, complementary with hard tissue histology a combined approach of clinical/CBCT examination, with nano-CT and histological mapping measurements, can provide an ideal platform for ECR imaging and exploration	Mavridou et al. [77]
understanding ECR patterns in endodontically treated teeth comparing characteristics/mechanisms of ECR in root filled teeth with those established in teeth with vital pulps	all endodontically treated teeth had similar ECR patterns patterns as an initiation, a resorption and a reparative stage differences between endodontically treated and teeth with vital pulps, mainly in the resorption and reparative stages resorption stage in root filled teeth more intense than repair stage, due to clastic cells and abundant granulation tissues possibly due to absence of pulp and protective PRRS layer and/or to altered chemical composition of root dentine after root canal treatment at repair stage, formation of reparative bonelike tissue took place to a lesser extent in root filled teeth	Mavridou et al. [78]
comparing phase transformation behavior, microstructure, nano-hardness, surface chemistry of HyFlex EDM instruments with conventional HyFlex CM	XRD analysis on HyFlex EDM revealed presence of martensite/rhombohedral R-phase, while martensite/austenite identified in HyFlex CM DSC analysis disclosed higher austenite finish temperatures for EDM instruments significant differences in nano-hardness/elasticity modulus between EDM/CM files FE-SEM and EDS analyses confirmed both EDM/CM files covered by an oxide layer rutile-TiO_2_ presence by micro-Raman spectroscopy HyFlex EDM revealed peculiar structural properties (increased phase transformation temperatures, hardness) enhanced mechanical behavior of instruments	Iacono et al. [79]
achieving a better understanding of a calcified extraradicular deposit on apical root surfaces of a mandibular first molar (radicular cyst/sinus tract) application of multimodular approach using a combination of multiple investigation methods	calcified extraradicular deposit can develop on the apical root surfaces of teeth with apical periodontitis in association with a radicular cyst/sinus tract sinus tract serves as the only communication between the apical lesion and the oral cavity whilst no periodontal defects present intra-oral radiography, high-resolution CBCT, nano-CT, hard tissue histology, EPMA can reveal calculus-like appearance and composition of extraradicular deposit calcified extraradicular deposits appear hyperechoic on ultrasound imaging and lead to occurrence of twinkling artefacts due to their rough mineralized surface	Petitjean et al. [80]
review of aetiology, potential predisposing factors, histopathology, diagnosis of ECR	several potential predisposing factors identified for ECR; certain combinations of factors result in a higher frequency of ECR more research required to confirm the cause and effect relationship most commonly affected teeth appear as maxillary incisor, canine, first molar and mandibular first molar teeth three stages in the process of ECR; initiation, progression/resorption and reparative phase. Resorption and repair/remodeling progress in parallel at different areas of affected tooth increased accuracy of CBCT results in more accurate detection, assessment of ECR, selection of the most appropriate treatment plan	Patel et al. [74]
introducing a methodology to simultaneously visualize dental ultrastructures (cellular/soft tissue components) by utilizing PTA as a contrast-enhancement agent	dental cementum/periodontium/odontoblasts/predentine made visible by PTA staining in high-resolution 3D nano-CT scans different segments of tooth required different staining protocols thickness of cementum computed over length of tooth making visible by PTA-enhanced contrast, attached soft tissue components of the interior tooth shown on the dentine–pulp interface in greater detail 3D illustrations allowed a histology-like visualization of sections in all orientations with a single scan/easy sample preparation 3D and quantitative analysis of dentine composition (tubular lumen, ratio of tubular lumen area to the dentinal surface) by segmentation of sigmoidal dentinal tubules and surrounding dentine visualization of hard tissues along with cellular layers/soft tissues in teeth using a laboratory-based nano-CT technique by staining protocol the protocol depended on tissue type/size methodology offered enhanced possibilities for concomitant visualization of soft/hard dental tissues	Hildebrand et al. [81]
evaluating volumes of total obturation and voids in different obturation techniques using nano-CT imaging	obturation technique and materials used significantly affect total volume of obturation material and voids	Holmes et al. [82]
